# Supplementary material for: Factors associated with mortality of the elderly due to ambulatory care sensitive conditions, between 2008 and 2018, in the Federal District, Brazil
Source: PLoS One. 2022 Aug 5;17(8):e0272650. doi: 10.1371/journal.pone.0272650 (PMC9355228; doi:10.1371/journal.pone.0272650)
Supplement: Attachment — Submitted filename: PONE-D-21-36313_reviewer.pdf [file pone.0272650.s003.pdf]

PLOS ONE

FACTORS ASSOCIATED WITH MORTALITY OF THE ELDERLY DUE TO AMBULATORY CARE SENSITIVE CONDITIONS, BETWEEN 2008 AND 2018, IN THE FEDERAL DISTRICT, BRAZIL

--Manuscript Draft--

**Manuscript Number:**	PONE-D-21-36313
**Article Type:**	Research Article
**Full Title:**	FACTORS ASSOCIATED WITH MORTALITY OF THE ELDERLY DUE TO AMBULATORY CARE SENSITIVE CONDITIONS, BETWEEN 2008 AND 2018, IN THE FEDERAL DISTRICT, BRAZIL
**Short Title:**	FACTORS ASSOCIATED WITH MORTALITY OF THE ELDERLY, BETWEEN 2008 AND 2018, BRAZIL
**Corresponding Author:**	Geraldo Marques da Costa<br>Universidade de Brasilia<br>Brasília- DF, Brasil, BRAZIL
**Keywords:**	Aged; Mortality; Primary Health Care; housing
**Abstract:**	Introduction<br><br>In Brazil, the Unified Health System (Sistema Único de Saúde, or SUS) provides health care, and aging population overwhelms the system due to the greater vulnerability of the elderly. In the Federal District, two models of primary care coexist – the traditional primary care and the family health strategy. The present study aimed to analyze the factors associated with mortality of the elderly due to conditions sensitive to ambulatory care in the Federal District, Brazil.<br><br>Methods<br><br>This time series study investigated all deaths that occurred in people over 60 years old between 2008 and 2018. The variables studied were age at death, sex, marital status, education, race/color, death by condition sensitive to ambulatory care, population coverage of primary care services. The Urban Well-Being Index (UWBI) was used, which includes the dimensions: mobility, environmental and housing conditions, infrastructure, and collective services, to analyze issues related to the place where the senior citizen resides.<br><br>Results<br><br>The deaths 85,835 senior citizens were recorded during the study period. The factors associated with mortality in the elderly due to ambulatory care sensitive conditions were male, lower income, and less education. Residing in a place with poor UWBI presented a response gradient with higher mortality. Increased ambulatory care coverage was also associated with lower mortality.<br><br>Conclusions<br><br>The study evidenced an association between male gender, age, income, and education, and UWBI with lower mortality due to ambulatory care sensitive conditions, and these associations presented a response gradient. The study also found that increased coverage of the elderly population was associated with lower mortality from sensitive conditions. Keywords: Aged; Mortality; Primary Health Care; Housing
**Order of Authors:**	Geraldo Marques da Costa
	Mauro Niskier Sanchez
	Helena Eri Shimizu
**Additional Information:**	

Powered by Editorial Manager® and ProduXion Manager® from Aries Systems Corporation

<table>
<tr><th>Question</th><th>Response</th></tr>
<tr><td>Financial Disclosure

Enter a financial disclosure statement that describes the sources of funding for the work included in this submission. Review the submission guidelines for detailed requirements. View published research articles from PLOS ONE for specific examples.

This statement is required for submission and will appear in the published article if the submission is accepted. Please make sure it is accurate.

Unfunded studies
Enter: The author(s) received no specific funding for this work.

Funded studies
Enter a statement with the following details:
• Initials of the authors who received each award
• Grant numbers awarded to each author
• The full name of each funder
• URL of each funder website
• Did the sponsors or funders play any role in the study design, data collection and analysis, decision to publish, or preparation of the manuscript?
• NO - Include this sentence at the end of your statement: The funders had no role in study design, data collection and analysis, decision to publish, or preparation of the manuscript.
• YES - Specify the role(s) played.

* typeset</td><td>The author(s) received no specific funding for this swork</td></tr>
<tr><td>Competing Interests

Use the instructions below to enter a competing interest statement for this submission. On behalf of all authors, disclose any competing interests that could be perceived to bias this work—acknowledging all financial support and any other relevant financial or non-financial competing interests.</td><td>The authors have declared that no competing interests exist.</td></tr>
</table>

This statement is required for submission and **will appear in the published article** if the submission is accepted. Please make sure it is accurate and that any funding sources listed in your Funding Information later in the submission form are also declared in your Financial Disclosure statement.<br><br>View published research articles from *PLOS ONE* for specific examples.<br><br>**NO authors have competing interests**<br><br>Enter: *The authors have declared that no competing interests exist.*<br><br>**Authors with competing interests**<br><br>Enter competing interest details beginning with this statement:<br><br>*I have read the journal's policy and the authors of this manuscript have the following competing interests: [insert competing interests here]*<br><br>* typeset	
**Ethics Statement**<br><br>Enter an ethics statement for this submission. This statement is required if the study involved:<br><br>• Human participants<br>• Human specimens or tissue<br>• Vertebrate animals or cephalopods<br>• Vertebrate embryos or tissues<br>• Field research<br><br>Write "N/A" if the submission does not require an ethics statement.<br><br>General guidance is provided below. Consult the submission guidelines for detailed instructions. **Make sure that all information entered here is included in the Methods section of the manuscript.**	The project was approved by the Research Ethics Committee of the Faculty of Health at the University of Brasília, opinion nº 3,479,132; issued on July 31, 2019, Certificate of Presentation for Ethical Appraisal (CAAE) No. 08615719.3.0000.0030.

Powered by Editorial Manager® and ProduXion Manager® from Aries Systems Corporation

**Format for specific study types**<br><br>**Human Subject Research (involving human participants and/or tissue)**<br>• Give the name of the institutional review board or ethics committee that approved the study<br>• Include the approval number and/or a statement indicating approval of this research<br>• Indicate the form of consent obtained (written/oral) or the reason that consent was not obtained (e.g. the data were analyzed anonymously)<br><br>**Animal Research (involving vertebrate animals, embryos or tissues)**<br>• Provide the name of the Institutional Animal Care and Use Committee (IACUC) or other relevant ethics board that reviewed the study protocol, and indicate whether they approved this research or granted a formal waiver of ethical approval<br>• Include an approval number if one was obtained<br>• If the study involved *non-human primates*, add *additional details* about animal welfare and steps taken to ameliorate suffering<br>• If anesthesia, euthanasia, or any kind of animal sacrifice is part of the study, include briefly which substances and/or methods were applied<br><br>**Field Research**<br><br>Include the following details if this study involves the collection of plant, animal, or other materials from a natural setting:<br>• Field permit number<br>• Name of the institution or relevant body that granted permission	
**Data Availability**<br><br>Authors are required to make all data underlying the findings described fully available, without restriction, and from the time of publication. PLOS allows rare exceptions to address legal and ethical concerns. See the PLOS Data Policy and FAQ for detailed information.	Yes - all data are fully available without restriction

A Data Availability Statement describing where the data can be found is required at submission. Your answers to this question constitute the Data Availability Statement and **will be published in the article**, if accepted.<br><br>**Important:** Stating 'data available on request from the author' is not sufficient. If your data are only available upon request, select 'No' for the first question and explain your exceptional situation in the text box.<br><br>Do the authors confirm that all data underlying the findings described in their manuscript are fully available without restriction?	
**Describe where the data may be found in full sentences. If you are copying our sample text, replace any instances of XXX with the appropriate details.**<br><br>• If the data are **held or will be held in a public repository**, include URLs, accession numbers or DOIs. If this information will only be available after acceptance, indicate this by ticking the box below. For example: *All XXX files are available from the XXX database (accession number(s) XXX, XXX.*).<br>• If the data are all contained **within the manuscript and/or Supporting Information files**, enter the following: *All relevant data are within the manuscript and its Supporting Information files.*<br>• If neither of these applies but you are able to provide **details of access elsewhere**, with or without limitations, please do so. For example:<br><br>*Data cannot be shared publicly because of [XXX]. Data are available from the XXX Institutional Data Access / Ethics Committee (contact via XXX) for researchers who meet the criteria for access to confidential data.*<br><br>*The data underlying the results presented in the study are available*	All relevant data are within the manuscript and its Supporting Information files.

from (include the name of the third party and contact information or URL).<br>• This text is appropriate if the data are owned by a third party and authors do not have permission to share the data.<br><br>* typeset	
Additional data availability information:	

Manuscript Click here to access/download;Manuscript;Article Plos One nov 15.docx

FACTORS ASSOCIATED WITH MORTALITY OF THE ELDERLY DUE TO AMBULATORY CARE SENSITIVE CONDITIONS, BETWEEN 2008 AND 2018, IN THE FEDERAL DISTRICT, BRAZIL

Abstract

Introduction: In Brazil, the Unified Health System (Sistema Único de Saúde, or SUS) provides health care, and aging population overwhelms the system due to the greater vulnerability of the elderly. In the Federal District, two models of primary care coexist – the traditional primary care and the family health strategy. The present study aimed to analyze the factors associated with mortality of the elderly due to conditions sensitive to ambulatory care in the Federal District, Brazil.

Methods: This time series study investigated all deaths that occurred in people over 60 years old between 2008 and 2018. The variables studied were age at death, sex, marital status, education, race/color, death by condition sensitive to ambulatory care, population coverage of primary care services. The Urban Well-Being Index (UWBI) was used, which includes the dimensions: mobility, environmental and housing conditions, infrastructure, and collective services, to analyze issues related to the place where the senior citizen resides.

Results: The deaths 70.503 senior citizens were recorded during the study period. The factors associated with mortality in the elderly due to ambulatory care sensitive conditions were male, lower income, and less education. Residing in a place with poor UWBI presented a response gradient with higher mortality. Increased ambulatory care coverage was also associated with lower mortality.

Conclusions: The study evidenced an association between male gender, age, income, and education, and UWBI with lower mortality due to ambulatory care sensitive conditions, and these associations presented a response gradient. The study also found that increased coverage of the elderly population was associated with lower mortality from sensitive conditions.

Keywords: Aged, Mortality, Primary Health Care, Housing

INTRODUCTION

Due to the improved life expectancy, the increased number of senior citizens has pressured health systems for more efficient responses. In Brazil, to the Unified Health System (Sistema Único de Saúde, or SUS) is tasked with guarantee the right to health care, through health promotion, protection, and recovery procedures, considering the social determinants of health. The strong and growing link between socio-economic aspects and health continues to increase, particularly in the elderly population that has diverse vulnerabilities(1).

Good primary health care (PHC) helps to reduce hospitalizations and can impact the mortality of the elderly (2). PHC requires qualified professionals, service structure, and coordination with other levels of services.

In Brazil, PHC is configured in two models: the traditional primary care (TPC) consisting of specialist physicians (clinician, gynecologist, and pediatrician) and the Family Health Strategy (FHS) formed by a team with a doctor, nurse , nursing technician and community health agent who works in the area with a registered population of at most 4,000 people(3) .

Studies have shown that coverage by primary care contributes to the reduction of mortality in the population, including the elderly(4). Mortality from ambulatory care sensitive causes is an important performance indicator(5). In Brazil, Ordinance 221/2008 of the Ministry of Health created a list of avoidable hospitalizations by effective and/or accessible ambulatory care services in order to assess the quality of care provided; however, the PHC remains scantly available to the elderly population (6). Different countries have their own lists of ambulatory care sensitive conditions. A review showed a strong association between low socioeconomic status and risk of hospitalization for sensitive conditions, thus the relationship between ambulatory care and population morbidity is internationally evidenced(7).

It is known that other general socio-economic, cultural, and environmental factors influence the risk of dying in groups according to the levels of income, education, profession, sex, place of residence, and other factors related to mortality in the elderly (8). Worsening poverty and social exclusion has become increasingly evident, especially in large urban centers, with greater social inequalities, increasing extreme poverty and hunger(9). The issue of decent housing has been understood as a determinant of health and well-being, linked to patterns of illness, especially in communicable diseases, domestic accidents, and mental health(10).

Therefore, analysis in greater depth is needed on how these factors influence the mortality of the elderly due to ambulatory care sensitive conditions. This situation still needs further elucidation in the literature.

This study analyzed the factors associated with mortality in the elderly due to ambulatory care sensitive conditions in the Federal District, Brazil, from 2008 to 2018.

METHODS

The study is based on secondary data obtained from the base of the Health Department of the Federal District. Confidentiality and anonymity were observed in accordance with the Resolution of the National Health Council (CNS) nº 466, of December 12, 2012. The project was approved by the Research Ethics Committee of the Faculty of Health at the University of Brasília, opinion nº 3,479,132; issued on July 31, 2019, Certificate of Presentation for Ethical Appraisal (CAAE) No. 08615719.3.0000.0030. The ethics committee waived the requirement for informed consent.

This is a time-series study, based on mortality data of senior citizens over 60 years old, from 2008 to 2018 in the Federal District, Brazil. In Brazil, a senior citizen is defined as an individual aged 60 years or over (11).

The Federal District is in the Central Plateau of Brazil, located in the Center-Western region. The population in 2019 was estimated at 3,012,718, of which 328,379 were senior citizens(12).

The population studied consisted of all deaths that occurred in the Federal District in people 60 years old or more, between the years 2008 to 2018. Records referring to senior citizens who did not reside in the Federal District, or those with incomplete records were excluded.

Information on coverage of Primary Health Care was obtained from the Ministry of Health's website for information and management of primary care is www.egestorab.saude.gov.br. Coverage data were standardized using as a reference the months of December of each year of the analyzed historical series from 2008–2018.

The variables studied were: age at death (60–69, 70–79, >80), sex (male and female), marital status (single, married, widowed, separated/divorced, not stated), education (none, 0–3, 4–7, 8–11, >12 years of schooling), race/color (white, black, brown, Asian, indigenous, not stated) death by ambulatory care sensitive conditions, coverage by TPC and FHS.

To analyze the issues related to the senior citizen's place of residence, the Urban Well-Being Index (UWBI) was used. This index is composed of five dimensions: mobility, environmental conditions, housing conditions, infrastructure, and collective services. Each of these dimensions is calculated, then the arithmetic mean is calculated. The result is a value ranging from 0 to 1, in which the UWBI is better the closer to 1 and worse the closer to 0 (13).

The diseases were classified as ambulatory care sensitive conditions according to the ordinance 2008 of 221 of the Ministry of Health (14). The tenth version of the International Classification of Diseases (ICD), known as the ICD-10, was used.

The statistical analysis of the data initially relied on the bivariate analysis of the variables of interest to the study the outcome (death by sensitive condition). In the next step, a multivariate logistic regression analysis was performed (15), which was built according to a hierarchical structure of three blocks (distal, medial, and proximal). The adjustment variables were sex and age group variables for the model as a whole but were not restricted to just one of the considered levels.

The first block (distal) included the income group, which also represents a regional characterization for the regions in the Federal District. The second block (medial) has the expression variables of urban well-being, expressed by the UWBI composite index, together with coverage of primary care (TPC) and family health teams (FHS). The third block contained education, marital status, and race/color, which are proximal characteristics.

Statistical analyzes of data were performed using the R (16)v program. 2.2.5019 R Core Team, 2019.

RESULTS

There were 70.503 deaths among the elderly living in the Federal District between 2008 and 2018. Most deaths occurred in women (50.2%), with older seniors predominating (40.7%). Married individuals (38.4%) predominated, followed by widowers (31,3%). Most deaths occurred in seniors who were declared white (55,1%). A significant percentage of deaths resulted from ambulatory care sensitive conditions (29.2%)

Table 1 – Distribution of deaths of the elderly according to sociodemographic characteristics and ambulatory care sensitive conditions. Federal District, Brazil, 2008 to 2018.

		**N° of deaths**	**%**
Sex	Female	35.402	50.2
	Male	35.101	49.8
Age group	60 to 69 years old	18.993	26.9
	70 to 79 years old	22.820	32.4
	80 years old or above	28.690	40.7
Marital status	Single	12.467	17.7
	Married	27.105	38.4
	Widowed	22.052	31.3
	Separated/divorced	6.920	9.8
	Not indicated	1.959	2.8
Race/color	White	38.816	55.1
	Black	4.520	6.4
	Asian	437	0.6
	Brown	26.179	37.1
	Indigenous	43	0.1
	Not indicated	508	0.7
Sensitive	Yes	20.606	29.2
Condition	No	49.897	70.8

Source: Database of the Federal District Health Department

In Table 2, deaths in the elderly due to ambulatory care sensitive conditions were correlated with several factors. Males had a higher risk of dying from sensitive conditions. The data on the age group, adjusted for age, elucidated a gradient of greater risk of dying as the seniors age increased. Adjusted for sex and age, seniors with lower income exhibited a higher risk of death, but lower middle income had a slightly higher risk of dying than low income.

For the UWBI, when adjusted for sex, age, and income, a dose dependent response was observed – the worse the UWBI, the greater the risk of the senior dying due to sensitive conditions. An even lower risk of dying was noted related to primary care coverage, both in traditional TPC and in the FHS. The race/color of the senior, adjusted for sex, age, income, UWBI, and primary care coverage, did not obtain statistical significance in terms of the risk of dying. In the analysis of marital status, being married had a protective effect on the risk of dying for the elderly from

sensitive conditions. Education had a dose-response effect – the lower the education level, the higher the risk of dying from the studied causes.

Table 2- Hierarchical model: Factors associated with deaths of the elderly from ambulatory care sensitive conditions, Distrito Federal, Brazil. 2008–2018

Independent variables		Total	Sensitive condition		Model A (Block 1)				Model B (Block 2)				Model C (Block 3)			
			Yes	No		95% confidence interval				95% confidence interval				95% confidence interval		
					Odds ratio	Upper limit	Lower limit	P-value	Odds ratio	Upper limit	Lower limit	P-valor	Odds ratio	Upper Limit	Lower limit	P-valor
Sex	Female	35402	10953	24449	1.133	1.094	1.173	<0.001	1.135	1.095	1.175	<0.001	1.051	1.011	1.092	0.012
	Male (ref.)	35101	9653	25448												
Age group (years)	60 to 69 (ref.)	18993	4431	14562												
	70 to 79	22820	6615	16205	1.378	1.314	1.444	<0.001	1.380	1.316	1.447	<0.001	1.332	1.270	1.398	<0.001
	80 and over	28690	9560	19130	1.775	1.696	1.857	<0.001	1.794	1.714	1.877	<0.001	1.650	1.571	1.733	<0.001
Income group	High (ref.)	13003	3010	9993												
	Upper middle	27206	7886	19320	1.435	1.363	1.511	<0.001								
	Lower middle	26075	8387	17688	1.762	1.672	1.856	<0.001								
	Lower	3935	1235	2700	1.748	1.605	1.903	<0.001								
UWBI*	Very good (ref.)	11605	2654	8951												
	Good	8951	2355	6596					1.148	1.052	1.254	0.002				
	Average	25434	7689	17745					1.341	1.216	1.480	<0.001				
	Bad	23906	7718	16188					1.429	1.269	1.609	<0.001				
	Terrible	323	102	221					1.550	1.168	2.057	0.002				
FHS Coverage**	%								0.995	0.991	0.999	0.011				
TPC Coverage ***	%								0.996	0.994	0.999	0.001				
Race/color	White (ref.)	38816	11045	27771												
	Black	4520	1440	3080									1.067	0.992	1.148	0.080
	Brown	26179	7833	18346									1.007	0.968	1.047	0.736
	Others	480	149	331									1.048	0.841	1.304	0.678
Marital status	Single (ref.)	12467	3923	8544												
	Married	27105	7045	20060									0.862	0.819	0.908	<0.001
	Widowed	22052	7209	14843									0.993	0.943	1.046	0.786
	Separated/ legally divorced	6920	1858	5062									0.945	0.880	1.014	0.114
Education	None	13848	4912	8936									1.465	1.354	1.584	<0.001
	1 to 3 years	20462	6345	14117									1.324	1.232	1.423	<0.001

4 to 7 years	11638	3286	8352	1.216	1.128	1.311	<0.001
8 to 11 years	10255	2527	7728	1.134	1.053	1.221	0.001
12 years or more (ref.)	7922	1627	6295				

DISCUSSION

The present study found that being older and being male were factors associated with the mortality of senior citizens due to conditions sensitive to ambulatory care in the Federal District. These findings corroborate the results of other studies that showed a higher risk of dying among elderly men, who tend to have worse health indicators, probably due to their low adherence to preventive measures, and the consequent reduction in autonomy for activities of daily living and less involvement with community activities (17)(18)(19)

When the marital status of the senior citizens who passed away was analyzed, being married had a protective effect against death due to conditions sensitive to ambulatory care. Living with a spouse reduces the mortality of the elderly(20). This effect can be explained by better financial conditions, better education, and premarital conditions observed in married elderly(21).

Income conditions increased the risk of seniors dying from conditions sensitive to ambulatory care. Poverty, as well as the deprivations resulting from it, has worsened in recent years, compromising the survival of the elderly(18). In Japan, this situation was related to thousands of premature deaths(22). In the state of Pará, a study observed that the purchasing power of the elderly as well as access to work and income were associated with greater survival of senior citizens and should be observed and promoted by public health policies (23).

A gradient effect was observed, in that mortality of the elderly due to sensitive conditions decreased over the studied years. Higher education levels contribute to better self-care in the elderly, in addition to facilitating therapeutic adherence and understanding of health guidelines (24).

For the UWBI, when adjusted for sex, age, and income, a dose-response effect was observed in this study. The worse their UWBI, the greater their risk of dying from sensitive conditions. The data indicate that the great regional inequalities in the Federal District affect the elderly, which include the poor housing, mobility, and environmental conditions as well as collective services. A study of the Federal District showed that the UWBI ranged from 0.26 to 0.97, and 19% of the regions had poor UVBI, while 6% were classified as very poor(13). Senior citizens in these regions live in very precarious conditions, in informal housing, on unpaved streets, without sewage and running water, and with pollution. As shown by other studies, these conditions lead to several diseases, increasing the early mortality of the elderly (25)(26)(27).

The coverage of the population by primary care exhibited a protective effect on the mortality of the elderly due to sensitive conditions. The Federal District has had a significant increase in the number of family health teams in recent years, especially through the 2017 policy of converting traditional TPC teams to FHS (28). In addition to increasing coverage, this policy focused on improving the care model, through investments in professional training and monitoring (29) and greater capacity to offer comprehensive and holistic care to all segments of the population, including the elderly. The family health teams benefited from the *Programa Mais Médico* (more doctors), which alleviated the problem of the chronic lack of doctors in peripheral regions and other areas with severe poverty (30).

Several studies reiterate that the good coverage of primary care facilitates access to medical appointments, complementary exams, and the dispensing of medications, which helps reduce mortality of the vulnerable elderly population, especially due to complications of chronic diseases (31)(32)(33).

Mortality studies can be impacted by the quality reporting of the causes of death. Furthermore, the modeling of this study did not permit certification that the deceased elderly person used the primary health care service. In addition, income was estimated based on the place of residence, which may not correspond to the individual reality of the senior citizens investigated. Another limiting factor for the analysis was the change in the primary care model in 2017, which occurred near the end of the period studied.

CONCLUSIONS

The study evidenced an association between the sex, age, income, and education with mortality from ambulatory care sensitive conditions, with the strongest association between poor UWBI and the risk of dying in the elderly. These associations showed a response gradient. This finding indicates the need for public investments to improve urban living conditions to help reduce the death rate of this population. The study also found that increased PHC coverage of the elderly population was associated with lower mortality from sensitive conditions. Therefore, public policies to expand and strengthen primary care have the potential to protect and prolong the lives of the elderly.

REFERENCES

1. Geib LTC. Social determinants of health in the elderly. Cien Saude Colet [Internet]. 2012 Jan [cited 2021 Aug 31];17(1):123–33. Available from: http://www.scielo.br/j/csc/a/fbHvqCDM5Hcx5VKY3SXXXjP/abstract/?lang=en https://doi.org/10.1590/S1413-81232012000100015

2. Edwards ST, Peterson K, Chan B, Anderson J, Helfand M. Effectiveness of Intensive Primary Care Interventions: A Systematic Review [Internet]. Vol. 32, Journal of General Internal Medicine. Springer New York LLC; 2017 [cited 2021 Mar 8]. p. 1377–86. Available from: /pmc/articles/PMC5698228/ DOI: 10.1007/s11606-017-4174-z

3. Facchini LA, Tomasi E, Dilélio AS. Quality of Primary Health Care in Brazil: advances, challenges and perspectives. Saúde em Debate [Internet]. 2018 Sep [cited 2021 Sep 17];42(spe1):208–23. Available from: http://www.scielo.br/j/sdeb/a/TmzJ4T4MkCxFxbxpxTFXJsd/?lang=en https://doi.org/10.1590/0103-11042018S114

4. Partanen VM, Arffman M, Manderbacka K, KeskimakiI I. Mortality related to ambulatory care sensitive hospitalisations in Finland. Scand J Public Health [Internet]. 2020 Dec 1 [cited 2021 Aug 17];48(8):839–46. Available from: https://pubmed.ncbi.nlm.nih.gov/32755271/ DOI: 10.1177/1403494820944722

5. Rodrigues MM, Alvarez AM, Rauch KC. Trends in hospitalization and mortality for ambulatory care sensitive conditions among older adults. Rev Bras Epidemiol. 2019 Mar 14;22:e190010. Available from: https://www.scielo.br/j/rbepid/a/skTfn5KgN68k3D4Nntq4PPp/abstract/?lang=en https://doi.org/10.1590/1980-549720190010

6. Alfradique ME, Bonolo P de F, Dourado I, Lima-Costa MF, Macinko J, Mendonça CS, et al. Ambulatory care sensitive hospitalizations: elaboration of Brazilian list as a tool for measuring health system performance (Project ICSAP - Brazil). Cad Saude Publica [Internet]. 2009 Jun [cited 2019 May 9];25(6):1337–49. Available from: http://www.scielo.br/scielo.php?script=sci_arttext&pid=S0102-311X2009000600016&lng=pt&tlng=pt https://doi.org/10.1590/S0102-311X2009000600016

7. Wallar LE, Prophetis E De, Rosella LC. Socioeconomic inequalities in hospitalizations for chronic ambulatory care sensitive conditions: a systematic review of peer-reviewed literature, 1990–2018. Int J Equity Health [Internet]. 2020 May 4 [cited 2021 Aug 31];19(1). Available from: /pmc/articles/PMC7197160/

8. Stringhini S, Carmeli C, Jokela M, Avendano M, Muenning P, Guida F, et al. Socioeconomic status and the 25 × 25 risk factors as determinants of premature mortality: a multicohort study and meta-analysis of 1·7 million men and women. Lancet (London, England) [Internet]. 2017 Mar 25 [cited 2021 Jul 27];389(10075):1229–37. Available from: https://pubmed.ncbi.nlm.nih.gov/28159391/ DOI: 10.1016/S0140-6736(16)32380-7

9. Pitombeira DF, Oliveira LC de. Poverty and social inequality: tensions between rights and austerity and its implications for primary healthcare. Cien Saude Colet [Internet]. 2020 May 8 [cited 2021 Oct 6];25(5):1699–708. Available from: http://www.scielo.br/j/csc/a/hxkLHrVqkZ5Hy6ZVK9pZmtb/abstract/?lang=en

• https://doi.org/10.1590/1413-81232020255.33972019

10. Pasternak S. Housing and Health. Estud Avançados [Internet]. 2016 Jan 1 [cited 2021 Jul 31];30(86):51–66. Available from: http://www.scielo.br/j/ea/a/CHFxfxZBNt7kyDWRtSfXQFF/?lang=pt https://doi.org/10.1590/S0103-40142016.00100004

11. Mendes AM de OC, Robazzi ML do CC. The aged worker in contemporaneity. Rev Lat Am Enfermagem [Internet]. 2021 May 21 [cited 2021 Sep 17];29. Available from: http://www.scielo.br/j/rlae/a/jWjPmxVYKxKtv7VB5LHcbQt/?lang=en https://doi.org/10.1590/1518-8345.0000.3471

12. Pirâmide Etária – Info DF [Internet]. [cited 2021 Sep 17]. Available from: http://infodf.codeplan.df.gov.br/?page_id=6

13. Secretaria de Economia do Distrito Federal. O Índice de Bem-Estar Urbano (IBEU) do Distrito Federal [Internet]. 2020 [cited 2021 Aug 31]. Available from: http://www.codeplan.df.gov.br/wp-content/uploads/2018/02/NM-O-Índice-de-Bem-Estar-Urbano-IBEU-do-Distrito-Federal.pdf

14. Brasil. Ministério da Saúde. Portaria n. 221 de 17 de abril de 2008 .Lista Brasileira de Internações por Condições Sensíveis à Atenção Primária. Diário Oficial [da] República Federativa do Brasil, Brasília, DF, 2008. Available from: http://189.28.128.100/dab/docs/legislacao/portaria221_17_04_08.pdf

15. Kleinbaum DG, Klein M. Logistic Regression [Internet]. 3° Edição. New York, NY: Springer New York; 2010 [cited 2021 Aug 31]. (Statistics for Biology and Health). Available from: http://link.springer.com/10.1007/978-1-4419-1742-3

16. R: a language and environment for statistical computing [Internet]. [cited 2021 Aug 31]. Available from: https://www.gbif.org/pt/tool/81287/r-a-language-and-environment-for-statistical-computing

17. Sleeman KE, Brito M de, Etkind S, Nkhoma K, Guo P, Higginson IJ, et al. The escalating global burden of serious health-related suffering: projections to 2060 by world regions, age groups, and health conditions.Lancet Glob Heal [Internet]. 2019 Jul 1 [cited 2021 Aug 16];7(7):e883. Available from: /pmc/articles/PMC6560023/ DOI: 10.1016/S2214-

109X(19)30172-X

18. Bento JA, Santos JLF, Lebrãoz ML. Factors associated with the survival of elderly men in almost 15 years. Rev Bras Epidemiol [Internet]. 2021 May 28 [cited 2021 Aug 16];24. Available from: http://www.scielo.br/j/rbepid/a/FW3sbRW5QTwz6DNXt3vgGkN/abstract/?lang=pt https://doi.org/10.1590/1980-549720210021

19. Borim FSA, Francisco PMSB, Neri AL. Sociodemographic and health factors associated with mortality in community-dwelling elderly. Rev Saude Publica [Internet]. 2017 May 4 [cited 2021 Aug 16];51:42. Available from: https://doi.org/10.1590/S1518-8787.2017051006708 https://doi.org/10.1590/S1518-8787.2017051006708

20. Zueras P, Rutgliano R, Trias-Llimós S. Marital status, living arrangements, and mortality in middle and older age in Europe. Int J Public Health [Internet]. 2020 Jun 1 [cited 2021 Aug 16];65(5):627–36. Available from: https://pubmed.ncbi.nlm.nih.gov/32350551/ DOI: 10.1007/s00038-020-01371-w

21. Compton J, Pollak RA. The life expectancy of older couples and surviving spouses. PLoS One [Internet]. 2021 May 1 [cited 2021 Sep 17];16(5):e0250564. Available from: https://journals.plos.org/plosone/article?id=10.1371/journal.pone.0250564 DOI: 10.1371/journal.pone.0250564

22. Masashige S, Kondo N, Oshio T, Tabuchi T, Kondo K. Relative Deprivation, Poverty, and Mortality in Japanese Older Adults: A Six-Year Follow-Up of the JAGES Cohort Survey. Int J Environ Res Public Health [Internet]. 2019 Jan 2 [cited 2021 Aug 16];16(2). Available from: https://pubmed.ncbi.nlm.nih.gov/30634562/ DOI: 10.3390/ijerph16020182

23. Campos ACV, Gonçalves LHT. Aging demographic profile in municipalities in the state of Pará, Brazil. Rev Bras Enferm [Internet]. 2018 [cited 2021 Aug 16];71:591–8. Available from: http://www.scielo.br/j/reben/a/fdqr85ckhGss7njWfB5HTgq/abstract/?lang=pt https://doi.org/10.1590/0034-7167-2017-0070

24. Borba AK de OT, Arruda IKG, Marques AP de O, Leal MCC, Diniz A da S. Knowledge and attitude about diabetes self-care of older adults in primary health care. Cien Saude Colet. 2019;24(1):125–36. Available from: DOI: 10.1590/1413-81232018241.35052016

25. Silveira IH da, Junger WL. Green spaces and mortality due to cardiovascular diseases in the city of Rio de Janeiro Rev Saude Publica [Internet]. 2018 May 3 [cited 2021 Jul 31];52. Available from: http://www.scielo.br/j/rsp/a/rMH7xb33VC4ntnHStnSj3hh/abstract/?lang=pt https://doi.org/10.11606/S1518-8787.2018052000290

26. Crouse DL, Pinault L, Balram A, Hystad P, Peters PA, Chen H, et al. Urban greenness and mortality in Canada's largest cities: a national cohort study. Lancet Planet Heal [Internet]. 2017 Oct 1 [cited 2021 Aug 4];1(7):e289–97. Available from: https://pubmed.ncbi.nlm.nih.gov/29851627/ https://doi.org/10.1016/S2542-5196(17)30118-3

27. Bernstein SF, Rehkopf D, Tuljapurkar S, Horvitz CC. Poverty dynamics, poverty thresholds and mortality: An age-stage Markovian model. PLoS One [Internet]. 2018 May 1 [cited 2021 Jul 31];13(5). Available from: /pmc/articles/PMC5955488/ DOI: 10.1371/journal.pone.0195734

28. Corrêa DSRC, Moura AG de OM, Quito MV, Souza HM de, Versiani LM, Leuzzi S, et al. Reform movements in the Federal District Health Care System:conversion of the Primary Health Care assistance model. Cien Saude Colet [Internet]. 2019 Jun 27 [cited 2021 Sep 14];24(6):2031–41. Available from: http://www.scielo.br/j/csc/a/TdcFYRMKPNHpcbtHWBVNmRg/abstract/?lang=en https://doi.org/10.1590/1413-81232018246.08802019

29. Fonseca HLP da. The Health Reform of Brasília, Brazil. Cien Saude Colet [Internet]. 2019 Jun 1 [cited 2020 Oct 13];24(6):1981–90. Available from: http://www.scielo.br/scielo.php?script=sci_arttext&pid=S1413-81232019000601981&tlng=pt https://doi.org/10.1590/1413-81232018246.07902019

30. Mourão Netto JJ, Rodrigues ARM, Aragão OC, Goyanna NF, Cavalcante AES, Vasconcelos MAS, et al. Contributions of the Mais Médicos (More Doctors) physician

recruitment program for health care in Brazil: an integrative review Rev Panam Salud Pública [Internet]. 2018 Jun 7 [cited 2021 Sep 18];42:e2. Available from: https://doi.org/10.26633/RPSP.2018.2 https://doi.org/10.26633/RPSP.2018.2

31. Schenker M, Costa DH da. Advances and challenges of health care of the elderly population with chronic diseases in Primary Health Care. Cien Saude Colet [Internet]. 2019 May 2 [cited 2021 Aug 16];24(4):1369–80. Available from: http://www.scielo.br/j/csc/a/fjgYFRhV7s4Tgqvdf5LKBDj/abstract/?format=html&lang=pt https://doi.org/10.1590/1413-81232018244.01222019

32. Macinko J, Mendonça CS. The Family Health Strategy, a strong model of Primary Health Care that delivers results. Saúde em Debate [Internet]. 2018 Sep [cited 2020 Oct 13];42(spe1):18–37. Available from: http://www.scielo.br/scielo.php?script=sci_arttext&pid=S0103-11042018000500018&lng=pt&tlng=pt https://doi.org/10.1590/0103-11042018S102

33. Sawin G, O’Connor N. Primary Care Transformation [Internet]. Vol. 46, Primary Care - Clinics in Office Practice. W.B. Saunders; 2019 [cited 2020 Oct 20]. p. 549–60. Available from: https://pubmed.ncbi.nlm.nih.gov/31655751/ DOI: 10.1016/j.pop.2019.07.006

Manuscript Click here to access/download;Manuscript;Article Plos One nov 15.pdf

FACTORS ASSOCIATED WITH MORTALITY OF THE ELDERLY DUE TO AMBULATORY CARE SENSITIVE CONDITIONS, BETWEEN 2008 AND 2018, IN THE FEDERAL DISTRICT, BRAZIL

Abstract

Introduction: In Brazil, the Unified Health System (Sistema Único de Saúde, or SUS) provides health care, and aging population overwhelms the system due to the greater vulnerability of the elderly. In the Federal District, two models of primary care coexist – the traditional primary care and the family health strategy. The present study aimed to analyze the factors associated with mortality of the elderly due to conditions sensitive to ambulatory care in the Federal District, Brazil.

Methods: This time series study investigated all deaths that occurred in people over 60 years old between 2008 and 2018. The variables studied were age at death, sex, marital status, education, race/color, death by condition sensitive to ambulatory care, population coverage of primary care services. The Urban Well-Being Index (UWBI) was used, which includes the dimensions: mobility, environmental and housing conditions, infrastructure, and collective services, to analyze issues related to the place where the senior citizen resides.

Results: The deaths 70.503 senior citizens were recorded during the study period. The factors associated with mortality in the elderly due to ambulatory care sensitive conditions were male, lower income, and less education. Residing in a place with poor UWBI presented a response gradient with higher mortality. Increased ambulatory care coverage was also associated with lower mortality.

Conclusions: The study evidenced an association between male gender, age, income, and education, and UWBI with lower mortality due to ambulatory care sensitive conditions, and these associations presented a response gradient. The study also found that increased coverage of the elderly population was associated with lower mortality from sensitive conditions.

Keywords: Aged, Mortality, Primary Health Care, Housing

INTRODUCTION

Due to the improved life expectancy, the increased number of senior citizens has pressured health systems for more efficient responses. In Brazil, to the Unified Health System (Sistema Único de Saúde, or SUS) is tasked with guarantee the right to health care, through health promotion, protection, and recovery procedures, considering the social determinants of health. The strong and growing link between socio-economic aspects and health continues to increase, particularly in the elderly population that has diverse vulnerabilities(1).

Good primary health care (PHC) helps to reduce hospitalizations and can impact the mortality of the elderly (2). PHC requires qualified professionals, service structure, and coordination with other levels of services.

In Brazil, PHC is configured in two models: the traditional primary care (TPC) consisting of specialist physicians (clinician, gynecologist, and pediatrician) and the Family Health Strategy (FHS) formed by a team with a doctor, nurse , nursing technician and community health agent who works in the area with a registered population of at most 4,000 people(3) .

Studies have shown that coverage by primary care contributes to the reduction of mortality in the population, including the elderly(4). Mortality from ambulatory care sensitive causes is an important performance indicator(5). In Brazil, Ordinance 221/2008 of the Ministry of Health created a list of avoidable hospitalizations by effective and/or accessible ambulatory care services in order to assess the quality of care provided; however, the PHC remains scantly available to the elderly population (6). Different countries have their own lists of ambulatory care sensitive conditions. A review showed a strong association between low socioeconomic status and risk of hospitalization for sensitive conditions, thus the relationship between ambulatory care and population morbidity is internationally evidenced(7).

It is known that other general socio-economic, cultural, and environmental factors influence the risk of dying in groups according to the levels of income, education, profession, sex, place of residence, and other factors related to mortality in the elderly (8). Worsening poverty and social exclusion has become increasingly evident, especially in large urban centers, with greater social inequalities, increasing extreme poverty and hunger(9). The issue of decent housing has been understood as a determinant of health and well-being, linked to patterns of illness, especially in communicable diseases, domestic accidents, and mental health(10).

Therefore, analysis in greater depth is needed on how these factors influence the mortality of the elderly due to ambulatory care sensitive conditions. This situation still needs further elucidation in the literature.

This study analyzed the factors associated with mortality in the elderly due to ambulatory care sensitive conditions in the Federal District, Brazil, from 2008 to 2018.

METHODS

The study is based on secondary data obtained from the base of the Health Department of the Federal District. Confidentiality and anonymity were observed in accordance with the Resolution of the National Health Council (CNS) nº 466, of December 12, 2012. The project was approved by the Research Ethics Committee of the Faculty of Health at the University of Brasília, opinion nº 3,479,132; issued on July 31, 2019, Certificate of Presentation for Ethical Appraisal (CAAE) No. 08615719.3.0000.0030. The ethics committee waived the requirement for informed consent.

This is a time-series study, based on mortality data of senior citizens over 60 years old, from 2008 to 2018 in the Federal District, Brazil. In Brazil, a senior citizen is defined as an individual aged 60 years or over (11).

The Federal District is in the Central Plateau of Brazil, located in the Center-Western region. The population in 2019 was estimated at 3,012,718, of which 328,379 were senior citizens(12).

The population studied consisted of all deaths that occurred in the Federal District in people 60 years old or more, between the years 2008 to 2018. Records referring to senior citizens who did not reside in the Federal District, or those with incomplete records were excluded.

Information on coverage of Primary Health Care was obtained from the Ministry of Health's website for information and management of primary care is www.egestorab.saude.gov.br. Coverage data were standardized using as a reference the months of December of each year of the analyzed historical series from 2008–2018.

The variables studied were: age at death (60–69, 70–79, >80), sex (male and female), marital status (single, married, widowed, separated/divorced, not stated), education (none, 0–3, 4–7, 8–11, >12 years of schooling), race/color (white, black, brown, Asian, indigenous, not stated) death by ambulatory care sensitive conditions, coverage by TPC and FHS.

To analyze the issues related to the senior citizen's place of residence, the Urban Well-Being Index (UWBI) was used. This index is composed of five dimensions: mobility, environmental conditions, housing conditions, infrastructure, and collective services. Each of these dimensions is calculated, then the arithmetic mean is calculated. The result is a value ranging from 0 to 1, in which the UWBI is better the closer to 1 and worse the closer to 0 (13).

The diseases were classified as ambulatory care sensitive conditions according to the ordinance 2008 of 221 of the Ministry of Health (14). The tenth version of the International Classification of Diseases (ICD), known as the ICD-10, was used.

The statistical analysis of the data initially relied on the bivariate analysis of the variables of interest to the study the outcome (death by sensitive condition). In the next step, a multivariate logistic regression analysis was performed (15), which was built according to a hierarchical structure of three blocks (distal, medial, and proximal). The adjustment variables were sex and age group variables for the model as a whole but were not restricted to just one of the considered levels.

The first block (distal) included the income group, which also represents a regional characterization for the regions in the Federal District. The second block (medial) has the expression variables of urban well-being, expressed by the UWBI composite index, together with coverage of primary care (TPC) and family health teams (FHS). The third block contained education, marital status, and race/color, which are proximal characteristics.

Statistical analyzes of data were performed using the R (16)v program. 2.2.5019 R Core Team, 2019.

RESULTS

There were 70.503 deaths among the elderly living in the Federal District between 2008 and 2018. Most deaths occurred in women (50.2%), with older seniors predominating (40.7%). Married individuals (38.4%) predominated, followed by widowers (31,3%). Most deaths occurred in seniors who were declared white (55,1%). A significant percentage of deaths resulted from ambulatory care sensitive conditions (29.2%)

Table 1 – Distribution of deaths of the elderly according to sociodemographic characteristics and ambulatory care sensitive conditions. Federal District, Brazil, 2008 to 2018.

		N° of deaths	%
Sex	Female	35.402	50.2
	Male	35.101	49.8
Age group	60 to 69 years old	18.993	26.9
	70 to 79 years old	22.820	32.4
	80 years old or above	28.690	40.7
Marital status	Single	12.467	17.7
	Married	27.105	38.4
	Widowed	22.052	31.3
	Separated/divorced	6.920	9.8
	Not indicated	1.959	2.8
Race/color	White	38.816	55.1
	Black	4.520	6.4
	Asian	437	0.6
	Brown	26.179	37.1
	Indigenous	43	0.1
	Not indicated	508	0.7
Sensitive	Yes	20.606	29.2
Condition	No	49.897	70.8

Source: Database of the Federal District Health Department

In Table 2, deaths in the elderly due to ambulatory care sensitive conditions were correlated with several factors. Males had a higher risk of dying from sensitive conditions. The data on the age group, adjusted for age, elucidated a gradient of greater risk of dying as the seniors age increased. Adjusted for sex and age, seniors with lower income exhibited a higher risk of death, but lower middle income had a slightly higher risk of dying than low income.

For the UWBI, when adjusted for sex, age, and income, a dose dependent response was observed – the worse the UWBI, the greater the risk of the senior dying due to sensitive conditions. An even lower risk of dying was noted related to primary care coverage, both in traditional TPC and in the FHS. The race/color of the senior, adjusted for sex, age, income, UWBI, and primary care coverage, did not obtain statistical significance in terms of the risk of dying. In the analysis of marital status, being married had a protective effect on the risk of dying for the elderly from

sensitive conditions. Education had a dose-response effect – the lower the education level, the higher the risk of dying from the studied causes.

Table 2- Hierarchical model: Factors associated with deaths of the elderly from ambulatory care sensitive conditions, Distrito Federal, Brazil. 2008–2018

**Independent variables**		**Total**	**Sensitive condition**		**Model A (Block 1)**				**Model B (Block 2)**				**Model C (Block 3)**			
			Yes	No		95% confidence interval				95% confidence interval				95% confidence interval		
					Odds ratio	Upper limit	Lower limit	P-value	Odds ratio	Upper limit	Lower limit	P-valor	Odds ratio	Upper Limit	Lower limit	P-valor
Sex	Female	35402	10953	24449	1.133	1.094	1.173	<0.001	1.135	1.095	1.175	<0.001	1.051	1.011	1.092	0.012
	Male (ref.)	35101	9653	25448												
Age group (years)	60 to 69 (ref.)	18993	4431	14562												
	70 to 79	22820	6615	16205	1.378	1.314	1.444	<0.001	1.380	1.316	1.447	<0.001	1.332	1.270	1.398	<0.001
	80 and over	28690	9560	19130	1.775	1.696	1.857	<0.001	1.794	1.714	1.877	<0.001	1.650	1.571	1.733	<0.001
Income group	High (ref.)	13003	3010	9993												
	Upper middle	27206	7886	19320	1.435	1.363	1.511	<0.001								
	Lower middle	26075	8387	17688	1.762	1.672	1.856	<0.001								
	Lower	3935	1235	2700	1.748	1.605	1.903	<0.001								
UWBI*	Very good (ref.)	11605	2654	8951												
	Good	8951	2355	6596					1.148	1.052	1.254	0.002				
	Average	25434	7689	17745					1.341	1.216	1.480	<0.001				
	Bad	23906	7718	16188					1.429	1.269	1.609	<0.001				
	Terrible	323	102	221					1.550	1.168	2.057	0.002				
FHS Coverage**	%								0.995	0.991	0.999	0.011				
TPC Coverage ***	%								0.996	0.994	0.999	0.001				
Race/color	White (ref.)	38816	11045	27771												
	Black	4520	1440	3080									1.067	0.992	1.148	0.080
	Brown	26179	7833	18346									1.007	0.968	1.047	0.736
	Others	480	149	331									1.048	0.841	1.304	0.678
Marital status	Single (ref.)	12467	3923	8544												
	Married	27105	7045	20060									0.862	0.819	0.908	<0.001
	Widowed	22052	7209	14843									0.993	0.943	1.046	0.786
	Separated/ legally divorced	6920	1858	5062									0.945	0.880	1.014	0.114
Education	None	13848	4912	8936									1.465	1.354	1.584	<0.001
	1 to 3 years	20462	6345	14117									1.324	1.232	1.423	<0.001

4 to 7 years	11638	3286	8352	1.216	1.128	1.311	<0.001
8 to 11 years	10255	2527	7728	1.134	1.053	1.221	0.001
12 years or more (ref.)	7922	1627	6295				

DISCUSSION

The present study found that being older and being male were factors associated with the mortality of senior citizens due to conditions sensitive to ambulatory care in the Federal District. These findings corroborate the results of other studies that showed a higher risk of dying among elderly men, who tend to have worse health indicators, probably due to their low adherence to preventive measures, and the consequent reduction in autonomy for activities of daily living and less involvement with community activities (17)(18)(19)

When the marital status of the senior citizens who passed away was analyzed, being married had a protective effect against death due to conditions sensitive to ambulatory care. Living with a spouse reduces the mortality of the elderly(20). This effect can be explained by better financial conditions, better education, and premarital conditions observed in married elderly(21).

Income conditions increased the risk of seniors dying from conditions sensitive to ambulatory care. Poverty, as well as the deprivations resulting from it, has worsened in recent years, compromising the survival of the elderly(18). In Japan, this situation was related to thousands of premature deaths(22). In the state of Pará, a study observed that the purchasing power of the elderly as well as access to work and income were associated with greater survival of senior citizens and should be observed and promoted by public health policies (23).

A gradient effect was observed, in that mortality of the elderly due to sensitive conditions decreased over the studied years. Higher education levels contribute to better self-care in the elderly, in addition to facilitating therapeutic adherence and understanding of health guidelines (24).

For the UWBI, when adjusted for sex, age, and income, a dose-response effect was observed in this study. The worse their UWBI, the greater their risk of dying from sensitive conditions. The data indicate that the great regional inequalities in the Federal District affect the elderly, which include the poor housing, mobility, and environmental conditions as well as collective services. A study of the Federal District showed that the UWBI ranged from 0.26 to 0.97, and 19% of the regions had poor UVBI, while 6% were classified as very poor(13). Senior citizens in these regions live in very precarious conditions, in informal housing, on unpaved streets, without sewage and running water, and with pollution. As shown by other studies, these conditions lead to several diseases, increasing the early mortality of the elderly (25)(26)(27).

The coverage of the population by primary care exhibited a protective effect on the mortality of the elderly due to sensitive conditions. The Federal District has had a significant increase in the number of family health teams in recent years, especially through the 2017 policy of converting traditional TPC teams to FHS (28). In addition to increasing coverage, this policy focused on improving the care model, through investments in professional training and monitoring (29) and greater capacity to offer comprehensive and holistic care to all segments of the population, including the elderly. The family health teams benefited from the *Programa Mais Médico* (more doctors), which alleviated the problem of the chronic lack of doctors in peripheral regions and other areas with severe poverty (30).

Several studies reiterate that the good coverage of primary care facilitates access to medical appointments, complementary exams, and the dispensing of medications, which helps reduce mortality of the vulnerable elderly population, especially due to complications of chronic diseases (31)(32)(33).

Mortality studies can be impacted by the quality reporting of the causes of death. Furthermore, the modeling of this study did not permit certification that the deceased elderly person used the primary health care service. In addition, income was estimated based on the place of residence, which may not correspond to the individual reality of the senior citizens investigated. Another limiting factor for the analysis was the change in the primary care model in 2017, which occurred near the end of the period studied.

CONCLUSIONS

The study evidenced an association between the sex, age, income, and education with mortality from ambulatory care sensitive conditions, with the strongest association between poor UWBI and the risk of dying in the elderly. These associations showed a response gradient. This finding indicates the need for public investments to improve urban living conditions to help reduce the death rate of this population. The study also found that increased PHC coverage of the elderly population was associated with lower mortality from sensitive conditions. Therefore, public policies to expand and strengthen primary care have the potential to protect and prolong the lives of the elderly.

REFERENCES

1. Geib LTC. Social determinants of health in the elderly. Cien Saude Colet [Internet]. 2012 Jan [cited 2021 Aug 31];17(1):123–33. Available from: http://www.scielo.br/j/csc/a/fbHvqCDM5Hcx5VKY3SXXXjP/abstract/?lang=en https://doi.org/10.1590/S1413-81232012000100015

2. Edwards ST, Peterson K, Chan B, Anderson J, Helfand M. Effectiveness of Intensive Primary Care Interventions: A Systematic Review [Internet]. Vol. 32, Journal of General Internal Medicine. Springer New York LLC; 2017 [cited 2021 Mar 8]. p. 1377–86. Available from: /pmc/articles/PMC5698228/ DOI: 10.1007/s11606-017-4174-z

3. Facchini LA, Tomasi E, Dilélio AS. Quality of Primary Health Care in Brazil: advances, challenges and perspectives. Saúde em Debate [Internet]. 2018 Sep [cited 2021 Sep 17];42(spe1):208–23. Available from: http://www.scielo.br/j/sdeb/a/TmzJ4T4MkCxFxbxpxTFXJsd/?lang=en https://doi.org/10.1590/0103-11042018S114

4. Partanen VM, Arffman M, Manderbacka K, KeskimakiI I. Mortality related to ambulatory care sensitive hospitalisations in Finland. Scand J Public Health [Internet]. 2020 Dec 1 [cited 2021 Aug 17];48(8):839–46. Available from: https://pubmed.ncbi.nlm.nih.gov/32755271/ DOI: 10.1177/1403494820944722

5. Rodrigues MM, Alvarez AM, Rauch KC. Trends in hospitalization and mortality for ambulatory care sensitive conditions among older adults. Rev Bras Epidemiol. 2019 Mar 14;22:e190010. Available from: https://www.scielo.br/j/rbepid/a/skTfn5KgN68k3D4Nntq4PPp/abstract/?lang=en https://doi.org/10.1590/1980-549720190010

6. Alfradique ME, Bonolo P de F, Dourado I, Lima-Costa MF, Macinko J, Mendonça CS, et al. Ambulatory care sensitive hospitalizations: elaboration of Brazilian list as a tool for measuring health system performance (Project ICSAP - Brazil). Cad Saude Publica [Internet]. 2009 Jun [cited 2019 May 9];25(6):1337–49. Available from: http://www.scielo.br/scielo.php?script=sci_arttext&pid=S0102-311X2009000600016&lng=pt&tlng=pt https://doi.org/10.1590/S0102-311X2009000600016

7. Wallar LE, Prophetis E De, Rosella LC. Socioeconomic inequalities in hospitalizations for chronic ambulatory care sensitive conditions: a systematic review of peer-reviewed literature, 1990–2018. Int J Equity Health [Internet]. 2020 May 4 [cited 2021 Aug 31];19(1). Available from: /pmc/articles/PMC7197160/

8. Stringhini S, Carmeli C, Jokela M, Avendano M, Muenning P, Guida F, et al. Socioeconomic status and the 25 × 25 risk factors as determinants of premature mortality: a multicohort study and meta-analysis of 1·7 million men and women. Lancet (London, England) [Internet]. 2017 Mar 25 [cited 2021 Jul 27];389(10075):1229–37. Available from: https://pubmed.ncbi.nlm.nih.gov/28159391/ DOI: 10.1016/S0140-6736(16)32380-7

9. Pitombeira DF, Oliveira LC de. Poverty and social inequality: tensions between rights and austerity and its implications for primary healthcare. Cien Saude Colet [Internet]. 2020 May 8 [cited 2021 Oct 6];25(5):1699–708. Available from: http://www.scielo.br/j/csc/a/hxkLHrVqkZ5Hy6ZVK9pZmtb/abstract/?lang=en

• https://doi.org/10.1590/1413-81232020255.33972019

10. Pasternak S. Housing and Health. Estud Avançados [Internet]. 2016 Jan 1 [cited 2021 Jul 31];30(86):51–66. Available from: http://www.scielo.br/j/ea/a/CHFxfxZBNt7kyDWRtSfXQFF/?lang=pt https://doi.org/10.1590/S0103-40142016.00100004

11. Mendes AM de OC, Robazzi ML do CC. The aged worker in contemporaneity. Rev Lat Am Enfermagem [Internet]. 2021 May 21 [cited 2021 Sep 17];29. Available from: http://www.scielo.br/j/rlae/a/jWjPmxVYKxKtv7VB5LHcbQt/?lang=en https://doi.org/10.1590/1518-8345.0000.3471

12. Pirâmide Etária – Info DF [Internet]. [cited 2021 Sep 17]. Available from: http://infodf.codeplan.df.gov.br/?page_id=6

13. Secretaria de Economia do Distrito Federal. O Índice de Bem-Estar Urbano (IBEU) do Distrito Federal [Internet]. 2020 [cited 2021 Aug 31]. Available from: http://www.codeplan.df.gov.br/wp-content/uploads/2018/02/NM-O-Índice-de-Bem-Estar-Urbano-IBEU-do-Distrito-Federal.pdf

14. Brasil. Ministério da Saúde. Portaria n. 221 de 17 de abril de 2008 .Lista Brasileira de Internações por Condições Sensíveis à Atenção Primária. Diário Oficial [da] República Federativa do Brasil, Brasília, DF, 2008. Available from: http://189.28.128.100/dab/docs/legislacao/portaria221_17_04_08.pdf

15. Kleinbaum DG, Klein M. Logistic Regression [Internet]. 3° Edição. New York, NY: Springer New York; 2010 [cited 2021 Aug 31]. (Statistics for Biology and Health). Available from: http://link.springer.com/10.1007/978-1-4419-1742-3

16. R: a language and environment for statistical computing [Internet]. [cited 2021 Aug 31]. Available from: https://www.gbif.org/pt/tool/81287/r-a-language-and-environment-for-statistical-computing

17. Sleeman KE, Brito M de, Etkind S, Nkhoma K, Guo P, Higginson IJ, et al. The escalating global burden of serious health-related suffering: projections to 2060 by world regions, age groups, and health conditions.Lancet Glob Heal [Internet]. 2019 Jul 1 [cited 2021 Aug 16];7(7):e883. Available from: /pmc/articles/PMC6560023/ DOI: 10.1016/S2214-

109X(19)30172-X

18. Bento JA, Santos JLF, Lebrãoz ML. Factors associated with the survival of elderly men in almost 15 years. Rev Bras Epidemiol [Internet]. 2021 May 28 [cited 2021 Aug 16];24. Available from: http://www.scielo.br/j/rbepid/a/FW3sbRW5QTwz6DNXt3vgGkN/abstract/?lang=pt https://doi.org/10.1590/1980-549720210021

19. Borim FSA, Francisco PMSB, Neri AL. Sociodemographic and health factors associated with mortality in community-dwelling elderly. Rev Saude Publica [Internet]. 2017 May 4 [cited 2021 Aug 16];51:42. Available from: https://doi.org/10.1590/S1518-8787.2017051006708 https://doi.org/10.1590/S1518-8787.2017051006708

20. Zueras P, Rutgliano R, Trias-Llimós S. Marital status, living arrangements, and mortality in middle and older age in Europe. Int J Public Health [Internet]. 2020 Jun 1 [cited 2021 Aug 16];65(5):627–36. Available from: https://pubmed.ncbi.nlm.nih.gov/32350551/ DOI: 10.1007/s00038-020-01371-w

21. Compton J, Pollak RA. The life expectancy of older couples and surviving spouses. PLoS One [Internet]. 2021 May 1 [cited 2021 Sep 17];16(5):e0250564. Available from: https://journals.plos.org/plosone/article?id=10.1371/journal.pone.0250564 DOI: 10.1371/journal.pone.0250564

22. Masashige S, Kondo N, Oshio T, Tabuchi T, Kondo K. Relative Deprivation, Poverty, and Mortality in Japanese Older Adults: A Six-Year Follow-Up of the JAGES Cohort Survey. Int J Environ Res Public Health [Internet]. 2019 Jan 2 [cited 2021 Aug 16];16(2). Available from: https://pubmed.ncbi.nlm.nih.gov/30634562/ DOI: 10.3390/ijerph16020182

23. Campos ACV, Gonçalves LHT. Aging demographic profile in municipalities in the state of Pará, Brazil. Rev Bras Enferm [Internet]. 2018 [cited 2021 Aug 16];71:591–8. Available from: http://www.scielo.br/j/reben/a/fdqr85ckhGss7njWfB5HTgq/abstract/?lang=pt https://doi.org/10.1590/0034-7167-2017-0070

24. Borba AK de OT, Arruda IKG, Marques AP de O, Leal MCC, Diniz A da S. Knowledge and attitude about diabetes self-care of older adults in primary health care. Cien Saude Colet. 2019;24(1):125–36. Available from: DOI: 10.1590/1413-81232018241.35052016

25. Silveira IH da, Junger WL. Green spaces and mortality due to cardiovascular diseases in the city of Rio de Janeiro Rev Saude Publica [Internet]. 2018 May 3 [cited 2021 Jul 31];52. Available from: http://www.scielo.br/j/rsp/a/rMH7xb33VC4ntnHStnSj3hh/abstract/?lang=pt https://doi.org/10.11606/S1518-8787.2018052000290

26. Crouse DL, Pinault L, Balram A, Hystad P, Peters PA, Chen H, et al. Urban greenness and mortality in Canada's largest cities: a national cohort study. Lancet Planet Heal [Internet]. 2017 Oct 1 [cited 2021 Aug 4];1(7):e289–97. Available from: https://pubmed.ncbi.nlm.nih.gov/29851627/ https://doi.org/10.1016/S2542-5196(17)30118-3

27. Bernstein SF, Rehkopf D, Tuljapurkar S, Horvitz CC. Poverty dynamics, poverty thresholds and mortality: An age-stage Markovian model. PLoS One [Internet]. 2018 May 1 [cited 2021 Jul 31];13(5). Available from: /pmc/articles/PMC5955488/ DOI: 10.1371/journal.pone.0195734

28. Corrêa DSRC, Moura AG de OM, Quito MV, Souza HM de, Versiani LM, Leuzzi S, et al. Reform movements in the Federal District Health Care System:conversion of the Primary Health Care assistance model. Cien Saude Colet [Internet]. 2019 Jun 27 [cited 2021 Sep 14];24(6):2031–41. Available from: http://www.scielo.br/j/csc/a/TdcFYRMKPNHpcbtHWBVNmRg/abstract/?lang=en https://doi.org/10.1590/1413-81232018246.08802019

29. Fonseca HLP da. The Health Reform of Brasília, Brazil. Cien Saude Colet [Internet]. 2019 Jun 1 [cited 2020 Oct 13];24(6):1981–90. Available from: http://www.scielo.br/scielo.php?script=sci_arttext&pid=S1413-81232019000601981&tlng=pt https://doi.org/10.1590/1413-81232018246.07902019

30. Mourão Netto JJ, Rodrigues ARM, Aragão OC, Goyanna NF, Cavalcante AES, Vasconcelos MAS, et al. Contributions of the Mais Médicos (More Doctors) physician

recruitment program for health care in Brazil: an integrative review Rev Panam Salud Pública [Internet]. 2018 Jun 7 [cited 2021 Sep 18];42:e2. Available from: https://doi.org/10.26633/RPSP.2018.2 https://doi.org/10.26633/RPSP.2018.2

31. Schenker M, Costa DH da. Advances and challenges of health care of the elderly population with chronic diseases in Primary Health Care. Cien Saude Colet [Internet]. 2019 May 2 [cited 2021 Aug 16];24(4):1369–80. Available from: http://www.scielo.br/j/csc/a/fjgYFRhV7s4Tgqvdf5LKBDj/abstract/?format=html&lang=pt https://doi.org/10.1590/1413-81232018244.01222019

32. Macinko J, Mendonça CS. The Family Health Strategy, a strong model of Primary Health Care that delivers results. Saúde em Debate [Internet]. 2018 Sep [cited 2020 Oct 13];42(spe1):18–37. Available from: http://www.scielo.br/scielo.php?script=sci_arttext&pid=S0103-11042018000500018&lng=pt&tlng=pt https://doi.org/10.1590/0103-11042018S102

33. Sawin G, O'Connor N. Primary Care Transformation [Internet]. Vol. 46, Primary Care - Clinics in Office Practice. W.B. Saunders; 2019 [cited 2020 Oct 20]. p. 549–60. Available from: https://pubmed.ncbi.nlm.nih.gov/31655751/ DOI: 10.1016/j.pop.2019.07.006

Click here to access/download
Supporting Information
Approval of Committee for Ethics in Research.pdf

Click here to access/download
Supporting Information
Supporting Information.doc

Click here to access/download
Supporting Information
Database_70503Deaths_2008to2018_plosone.csv

Click here to access/download
Supporting Information
Dictionary_70503Deaths_2008to2018.xlsx
