# Supplementary material for: Factors associated with mortality of the elderly due to ambulatory care sensitive conditions, between 2008 and 2018, in the Federal District, Brazil
Source: PLoS One. 2022 Aug 5;17(8):e0272650. doi: 10.1371/journal.pone.0272650 (PMC9355228; doi:10.1371/journal.pone.0272650)
Supplement: Attachment — Submitted filename: Response to reviewers.docx [file pone.0272650.s004.docx]

Response to Reviewers

Thank you for reviewing our article entitled “FACTORS ASSOCIATED WITH MORTALITY OF THE ELDERLY DUE TO AMBULATORY CARE SENSITIVE CONDITIONS, BETWEEN 2008 AND 2018, IN THE FEDERAL DISTRICT, BRAZIL”. The suggested changes were made and certainly helped substantially improve our work.

The answers are below:

1)	The database of deaths belonging to the Government Health Department of the Federal District was used and monitored by the technical team of the Epidemiological Surveillance. It is a robust database that has been using the same software since 1998. Technicians evaluate the death certificates received, and if the data are inconsistent, additional information is requested from the hospitals or health units where the declarations were filled out. In addition, the Federal District has Death Verification Services (DVS) where professionals evaluate bodies whose cause of death is not well understood to define the cause of death. DVS performs autopsies in the following situations: diseases with mandatory notification, cases without definition of the underlying cause of death, and deaths in less than 24 hours of hospital admission, without diagnosis. The technicians for the death database of the Federal District meet weekly to ensure completeness of the data.

2)	The choice of variables occurred after reviewing the database, correlating with the objectives of the article. Initially, the classic variables, such as sex, age, marital status, and race/color, were analyzed. We list the causes of death and indicate those that occurred due to conditions sensitive to ambulatory care, according to Ordinance No. 221 of April 17, 2008, by the Ministry of Health of Brazil. To correlate with primary care, we investigated the coverage by the health service, differentiating between the traditional primary care model and the family health strategy model according to the address of the deceased elderly person. We wanted to understand the importance of social inequalities in the deaths of the elderly. Once the deaths of the elderly in the Federal District were studied, different socioeconomic realities in the same geographic unit were analyzed. There are elderly people with high economic power, living in the best urban conditions, and very poor elderly people living in places with poor infrastructure. To understand the influence of socioeconomic factors, we correlated it with the Urban Well-Being Index (UWBI).

This index is composed of five dimensions: mobility, environmental, and housing conditions, infrastructure, and collective services. Each of these dimensions is calculated, and then the arithmetic mean is calculated. The result is a value that ranges from 0 to 1. The UWBI is better the closer it is to 1 and worse the closer it is to 0. The address of the deceased elderly person was considered. We reiterate that all analyzed variables are plausible and previously had a possible causal relationship.

As for the expected results, the primary researcher, who is a family and community physician and works in the poorest region of the Federal District, wanted, among other things, to understand how the conditions of the place of residence influence the mortality of the elderly. We hypothesized that the analysis would show that the inequalities, expressed in our article by the UWBI, impact the mortality of the elderly, as the main researcher has empirically observed this phenomenon in his routine of care.

3)	We chose to use the ecological variables primary care coverage and UWBI in our analysis. These variables were shown to be adequate for the hierarchical modeling of the article, since they analyze the context of the geographic, urban, and housing conditions of individuals. We indicate as a possible limitation of the study that this modeling can be impacted by the ecological bias. These variables in the article do not work as a period-time control.

4)	The work performed a hierarchical modeling. In this model, we used age at the distal level (block A). The age effect was tested at this level, and in fact showed an association between older age and higher mortality from conditions sensitive to ambulatory care (OR: 1.775, p<0.001). The age variable was used to adjust the following variables at the medial level (block B) and at the proximal level (block C) to ensure better reliability.

Our study analyzed a common outcome for all research participants, the death of the elderly. To answer the hypotheses of the study, the outcome was classified as a binary variable: death from a sensitive condition – yes or no. The categorization of the outcome variable as binary would make the modeling resulting from counting unfeasible. Thus, we believe that the hierarchical model is more adequate for our objective.

Excellent colocation by the reviewer; however, the modeling performed answered our research question. Nevertheless, if the analysis suggested by the editor is essential, we would be willing to carry it out.

5) We made the change suggested by the editor, and we changed it to chance (odds).

We await the final answer and are available for any clarifications.

Sincerely.

The authors.
