# Supplementary material for: Factors associated with mortality of the elderly due to ambulatory care sensitive conditions, between 2008 and 2018, in the Federal District, Brazil
Source: PLoS One. 2022 Aug 5;17(8):e0272650. doi: 10.1371/journal.pone.0272650 (PMC9355228; doi:10.1371/journal.pone.0272650)
Supplement: Attachment — Submitted filename: Response to Reviewers.docx [file pone.0272650.s005.docx]

Response to Reviewers

Dear reviewers

Thank you for reviewing our article entitled “FACTORS ASSOCIATED WITH MORTALITY OF THE ELDERLY DUE TO AMBULATORY CARE SENSITIVE CONDITIONS, BETWEEN 2008 AND 2018, IN THE FEDERAL DISTRICT, BRAZIL”. PONE-D-21-36313R1 The suggested changes were answered and definitely contributed to substantially improve our work.

The answers are as follows:

**1- Coverage and urban well-being do not vary by individual, they only vary by period of time. What is the limitation of doing this? Does it act as a control for period?**

We appreciate the comment. We do not see any major limitation in the use of variables that may have changed over the period covered by the study, since for each death we associate what we believe to be the most appropriate value for that point in time. Well-being has only one measure for the period, so we had no choice of which value to use for each event. Primary Care coverage was defined for each death considering the address where the elderly person resided, who was covered by either Traditional Primary Care or Family Health Strategy in December of each year of the study period. This study was cross-sectional, and the variables mentioned were not included in order to control for the period, but rather to assess whether changes in their levels lead to an increase or decrease in the chance that the outcome of interest occurs. As it is not possible at the ecological level to have different values of these factors at the same moment in time, the only way to assess their effects was to analyze the set of deaths over several years, within the proposed methodological design.

**2- Mortality increases with age - there are more deaths at older ages than any other age. Thus, it is expect that we see deaths increasing with your age in the analysis. One alternative is to model age specific mortality rates. One can obtain population by sex and age from IBGE or other sources. Then, you could model death rates or apply a poisson model on the counts of death. I believe modeling only death counts without a exposure measure limits the discussion of the results and might be affecting your results.**

As noted by the reviewer, we believed that age could impact the chance of dying. To adjust for the effect of age, it was included in the distal level of the hierarchical model, which allows us to interpret all coefficients at the same level and at subsequent levels (intermediate and proximal) as independent effects of the variables, already adjusted for age. Our objectives were to investigate possible predictors of dying from ambulatory care sensitive conditions in the age group of the study. For this reason, we opted for logistic regression (binary outcome) with the hierarchical approach already mentioned.

As indicated in the answer to item 1, this is a cross-sectional study, and the correction is already explained in the materials and methods section.

We appreciate the considerations and are available to conduct any additional analyzes requested by the reviewer.

We await the final answer and are available for any clarifications.

Sincerely.

The authors.
